# Validation of an interprofessional education search strategy in PubMed to optimize IPE literature searching

**DOI:** 10.5195/jmla.2024.1742

**Published:** 2024-01-16

**Authors:** Rebecca Carlson, Sophie Nachman, Lisa de Saxe Zerden, Nandita Mani

**Affiliations:** 1 rcarlson@unc.edu, Health Sciences Librarian and Liaison to the Eshelman School of Pharmacy, University of North Carolina at Chapel Hill, NC; 2 asophie@live.unc.edu, Graduate Assistant, Health Sciences Library; Master of Public Health student, Gillings School of Global Public Health, University of North Carolina at Chapel Hill, NC; 3 lzerden@email.unc.edu, Associate Professor, School of Social Work, University of North Carolina at Chapel Hill, NC; 4 nsmani@umass.edu, Dean of University Libraries, University of Massachusetts Amherst, Amherst, MA

**Keywords:** Interprofessional education, search hedge validation, relative recall, systematic reviews as topic

## Abstract

**Objective:**

With exponential growth in the publication of interprofessional education (IPE) research studies, it has become more difficult to find relevant literature and stay abreast of the latest research. To address this gap, we developed, evaluated, and validated search strategies for IPE studies in PubMed, to improve future access to and synthesis of IPE research. These search strategies, or search hedges, provide comprehensive, validated sets of search terms for IPE publications.

**Methods:**

The search strategies were created for PubMed using relative recall methodology. The research methods followed the guidance of previous search hedge and search filter validation studies in creating a gold standard set of relevant references using systematic reviews, having expert searchers identify and test search terms, and using relative recall calculations to validate the searches' performance against the gold standard set.

**Results:**

The three recommended search hedges for IPE studies presented had recall of 71.5%, 82.7%, and 95.1%; the first more focused for efficient literature searching, the last with high recall for comprehensive literature searching, and the remaining hedge as a middle ground between the other two options.

**Conclusion:**

These validated search hedges can be used in PubMed to expedite finding relevant scholarships, staying up to date with IPE research, and conducting literature reviews and evidence syntheses.

## INTRODUCTION

For more than a half century, interprofessional education (IPE) has continued to gain traction across clinical practice settings, health-related and adjacent professions, educational institutions, professional organizations, accrediting bodies, and health systems broadly [1]. Defined by the World Health Organization (WHO), IPE occurs “when students from one or more professions learn about, from, and with each other to enable effective collaboration and improve health outcomes” [2]. Because IPE influences collaborative practice (IPECP) and affects many different disciplines, the literature base in the field has grown considerably, yet gaps persist in analyzing and assessing this literature [3,4]. For example, between 1970 and 2010, the number of IPE related research publications increased by more than 2,290% [5,6]. In 2013, the Institute of Medicine (IOM) held a Global Forum on Innovation in Health Professional Education that included two workshops on IPE. A core theme from these meetings centered on IPE and IPECP research and metrics. Specifically, forum conveners and participants asked: “What data and metrics are needed to evaluate the impact of IPE on individual, population, and systems outcomes?” [7] However, answering this question is near impossible without first understanding how to systematically search and optimize the vast amount of literature currently available.

Scholarship on IPE and IPECP is useful to share evidence on the efficacy of specific IPE activities and how these activities can be replicated or revised [4]. However, the IPE literature is multifaceted not just in content but also in terms of methodology, outcomes, and the literature databases in which it can be found. Common challenges when searching the IPE literature include changes in IPE terminology, the growing number of professions contributing to IPE literature, the intermingling of education and collaboration literature, and various outcome measures (i.e., learner skills, provider attitudes, population health outcomes), and varied methodological approaches [4,8,9]. As Kim and Lee [10] note, “existing literature analysis method requires a considerable labor force, and there are time, effort, and accuracy limitations when analyzing the breadth of IPE literature.” As the field of IPE continues to develop, considerations on how best to search for relevant literature and establish effective search strategies are necessary.

Given the growth of literature in IPE, it is increasingly difficult for researchers to maintain a comprehensive understanding of the most up-to-date evidence. One solution to this is the use of validated search hedges, or search strategies, created by librarians or other expert searchers. Search strategies subject to an objective assessment of search performance can improve consistency and reproducibility of literature searching [11,12]. Search hedges are defined as a set of predetermined search terms which have been tested for their effectiveness in retrieving a specific type of evidence or literature from bibliographic databases [11,12]. They are developed to improve ease and efficiency in finding literature [13]. Rather than individual researchers having to create ad hoc sets of search terms each time they need to find literature, using a pre-created search strategy can be more time effective. However, to ensure that these search hedges are high quality, it is necessary to formally test their performance through the validation process. Validated search hedges are frequently designed to find certain study types or methodologies [12,14] and have also been developed for topic areas such as evidence-based practice [15], health equity [16], and geographic locations [11,17].

There are several methods for designing and evaluating search hedges [12]. Generally, though, these methods follow four steps: “(1) search term selection; (2) identification of a 'gold standard'; (3) evaluation of the search filter; (4) validation” [12]. The so-called “gold standard” is a list of relevant references which are used to test the effectiveness of the search hedge. The identification of a gold standard can be through hand searching, a combination of hand searching and database searching, the use of an existing definitive collection, or the use of a composite collection. Relying on hand searching in whole or part is labor-intensive, so where an authoritative collection of relevant articles does not yet exist, the creation of a composite collection can be an efficient way to manage the process. This methodology, called the relative recall method for search hedge validation, was pioneered in 2006 [14] and has since been used by many other scholars [16–22].

Since there is no current, definitive collection of IPE literature to use as a gold standard set, the authors of this study chose to follow the relative recall method of creating a composite set of literature to use as the gold standard for the validation process. While this methodology is not new, it had not yet been applied to IPE scholarship and, to the best of the authors' knowledge, this is the first IPE search hedge validation study published. This paper will provide the first set of formally validated and recommended terms for finding IPE scholarship which may make future identification of IPE research more efficient.

## METHODS

### Creation of Gold Standard Set

To create the gold standard set of references against which the completed search hedge would be measured, the authors searched PubMed using the keywords “interprofessional OR interdisciplinary OR IPE”, limited to the title field, and including the database publication type filter for systematic reviews. This method of sourcing systematic reviews was designed to find reviews across all dates and disciplines to prevent creating a gold standard set biased toward any one discipline or time period (given the changes in IPE terminology over the years). The titles of the 152 retrieved results were screened by one author (RC) and 18 that were obviously irrelevant (e.g., animal studies) based on their titles were excluded. The remaining 134 results were imported into Covidence and two authors (RC and SN) independently screened titles and abstracts and then full text articles against pre-set eligibility criteria, to create an unbiased pool of reviews [16,19,23]. Screening conflicts were resolved via discussion and consensus.

The eligibility criteria for systematic reviews included in the development of the gold standard reference set was twofold; to be selected, the papers needed to focus on IPE and to be high quality systematic reviews. High quality systematic reviews were defined as those that followed the PRISMA reporting guidelines and included a comprehensive literature search. To meet the inclusion criteria, the reviews needed to focus on IPE specifically and exclusively. This was defined as study populations that included two or more professions, an educational intervention or outcome and based on the WHO widely accepted definition of IPE. To be high quality, reviews needed PRISMA-compliant reporting of systematic review search methodology [24] in the methods section. Given the abundance of low-quality systematic reviews in the literature [25,26], the authors applied the criteria as generously as possible. Reviews lacking one or more of these inclusion criteria were excluded, including reviews partially but not exclusively focused on IPE. Also, if there were review updates published and the latest review included all citations from the previously published reviews, only the most recent review was considered for inclusion.

### Development of Search Strategies

The search hedges were developed in an iterative fashion by two literature searching experts. Keywords and subject headings were identified and tested from a range of sources: terms used in landmark IPE papers and other literature [2,4,5,7], search strategies used by Cochrane IPE reviews in the 2000s and early 2010s [27–29], and terms recommended by members of the research team. The first list of search terms for IPE was sent to an expert (in literature searching and IPE) not otherwise involved in any stage of the project. They peer reviewed the search terms using the PRESS guidelines and their recommendations were incorporated into the search design [30]. Other IPE experts on the research team also completed informal reviews of the search terms and gave input on search term inclusion.

The updated list of search terms was tested term by term for relevance and performance in PubMed. The authors reviewed search results when adding and removing search terms to identify their use in the literature and the number of papers added to the results and thus to determine which versions of the search should move forward to relative recall testing. Terms that did not add any unique results were excluded, to streamline the search hedges, and terms that did not add any results relevant to IPE were also excluded, to remove irrelevant citations. Different versions of the search strategy were created to test the performance of a phrase-based approach to the search versus individual keywords, the importance of a broad versus narrow interpretation of education terminology, and the best-suited PubMed field tags (e.g. [ti], [tiab], [tw]). Ultimately, each search term was tested multiple times to see the types of studies it returned before being included in the final search hedges and undergoing validation. The frequency of occurrence for each included IPE term was calculated using an internal tool [31]. The tool runs Python code to search for a list of keywords or phrases in a set of titles and abstracts. Results are output in Excel and include a count of how many times each term appears in the text corpus. Using this tool allows for greater efficiency when calculating term frequency for a large set of keywords and is the same methodology as could be carried out manually [31]. Occurrence data allowed authors to analyze changes in terminology over time (e.g., is early terminology for IPE such as “interdisciplinary education” still essential to use as a keyword in more recent publications). If the terms used in older publications were significantly different from the terms used in current publications, the authors intended to provide separate versions of the search; however, the older terms were determined to be still relevant for finding current publications and so were included in the final, formally tested search strategies.

### Recall and Relative Recall

Recall is the measurement of the proportion of available, relevant results in a database that a search hedge retrieves [13] and equals the number of relevant records retrieved by the search divided by the total number of relevant records [32]. The resulting number can be multiplied by 100 to then be expressed as a percentage. Relative recall is a measure of recall used in search hedge validation that measures the total number of articles retrieved by the search from the gold standard set [14,23]. Since researchers cannot know the total number of relevant records in a database without screening them all, they use relative recall to estimate the recall of the search hedge. Relative recall as a percentage is used to show the proportion of relevant articles retrieved by the search out of all the relevant articles available [12,14].



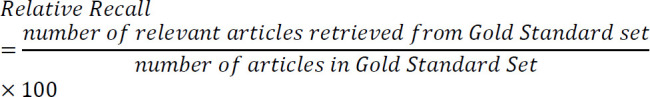



As such, this study used relative recall methodology to design and evaluate a search hedge for IPE literature.

### Relative Recall Validation Process

The relative recall calculations measured each search hedge against the gold standard set that was created from the IPE systematic reviews. Using the PubMed advanced search page, the team combined each search hedge with the gold standard set of articles and used the number of gold standard articles that appeared in each set of search results as the measurement of relative recall. These measurements were used to determine which search hedges performed the best, would be most useful, and should be included in the results.

**Figure 1:**
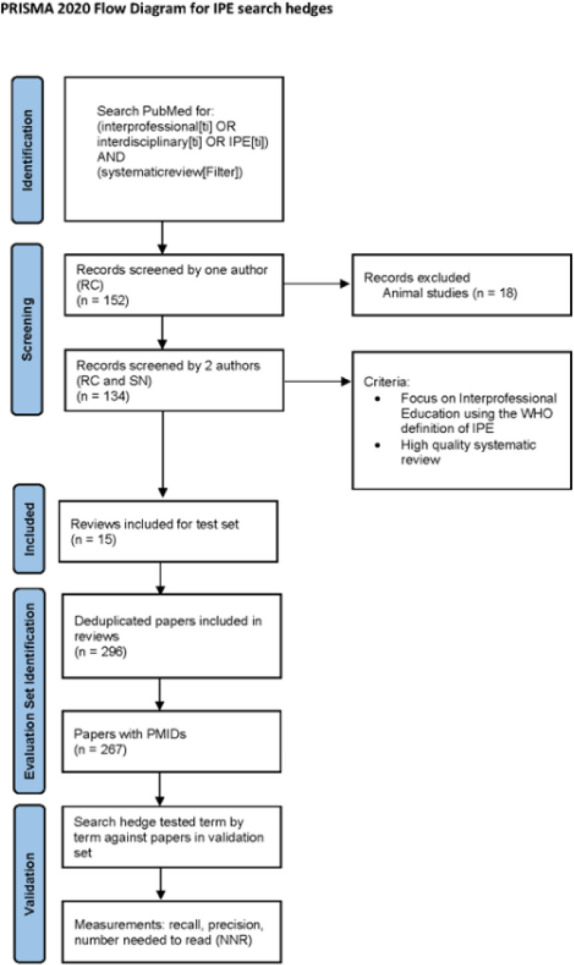
PRISMA flow diagram [24]

A PRISMA flow diagram (Fig. 1) shows the development process of the gold standard set and search hedges.

## RESULTS

### Gold Standard Set

After screening, there were 13 included systematic reviews published from 2008-2021 that covered various professions, levels of trainees, educational topics, and interventions across health sciences education. The 13 reviews contained 296 unique papers, 267 of which had PubMed identification numbers (PMIDs). Because this search was going to be validated in PubMed, only the papers with PMIDs (or papers indexed in PubMed) were included in the gold standard set so the relative recall measurements would be accurate. These 267 papers, published from 1981-2021, well exceed the minimum recommended number of 100 original papers for a search hedge validation set following relative recall methods [14] and cover a broad range of years up to current scholarship. This set of 267 papers became the gold standard set used for search strategy validation [33].

### Search Strategies

The sets of search terms presented here performed the best out of the 12 search strategies developed and tested, as they had high relative recall and can meet a range of research aims. Also, recommendations are provided for individual IPE search terms that have the highest frequency in the results and the best recall, to give additional search options beyond the full search hedges. These various PubMed search options will give IPE scholars objective data to choose the set of search terms that matches their aims and search for IPE literature in the way that best suits their needs. The searches as presented in Table 1 are intended to be copied and pasted directly into PubMed or included within a larger search strategy for ease of application.

**Table 1:** Search Hedges with Percent Recall and Total Results

Number	Search Title	Percent Recall	Total Results*
**#1**	Narrow Phrase Search: (IPE[tiab] OR “interprofessional education”[Mesh] OR “interprofessional education”[tw] OR “inter-professional education”[tw] OR “interdisciplinary education”[tw] OR “inter-disciplinary education”[tw] OR “multiprofessional education”[tw] OR “multidisciplinary education”[tw] OR “multi-professional education”[tw] OR “multi-disciplinary education”[tw])	71.5%	5,200
**#2**	Narrow Title Search: (interprofessional*[ti] OR inter-professional*[ti] OR multiprofessional*[ti] OR multi-professional*[ti] OR interdisciplinary[ti] OR inter-disciplinary[ti] OR multidisciplinary[ti] OR multi-disciplinary[ti] OR multioccupational[ti] OR interoccupational[ti] OR inter-occupational[ti]) AND (student*[ti] OR educat*[ti] OR learn*[ti] OR train*[ti] OR teach*[ti] OR curricul*[ti] OR simulat*[ti] OR school*[ti] OR course*[ti])	82.7%	6,555
**#3**	Broader Keyword Search: (IPE[tiab] OR “interprofessional education”[Mesh]) OR ((interprofessional*[tiab] OR interprofessional*[tiab] OR interdisciplinary[tiab] OR inter-disciplinary[tiab] OR multidisciplinary[tiab] OR “Interprofessional Relations” [Mesh]) AND (student[tiab] OR students[tiab] OR educate[tiab] OR educating[tiab] OR educator[tiab] OR educators[tiab] OR education[tiab] OR instructor[tiab] OR instructors[tiab] OR instruction[tiab] OR teaching[tiab] OR training[tiab] OR trainee[tiab] OR trainees[tiab] OR curriculum[tiab] OR curricula[tiab] OR simulation[tiab] OR simulations[tiab] OR shadowing[tiab] OR “clinical practicum*”[tiab] OR “clinical placement*“[tiab] OR “experiential learning”[tiab] OR teamwork[tiab] OR “Education, Professional”[Mesh] OR “Clinical Competence” [Mesh]))	95.13%	55,791

* **Result numbers as of August 26, 2022**

**Figure 2:**
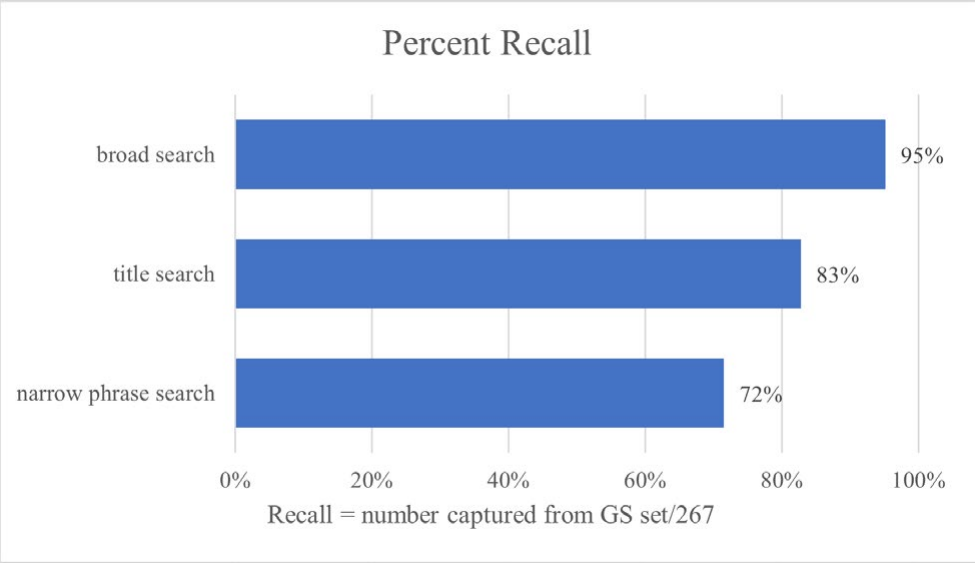
Search hedges and recall

**Figure 3:**
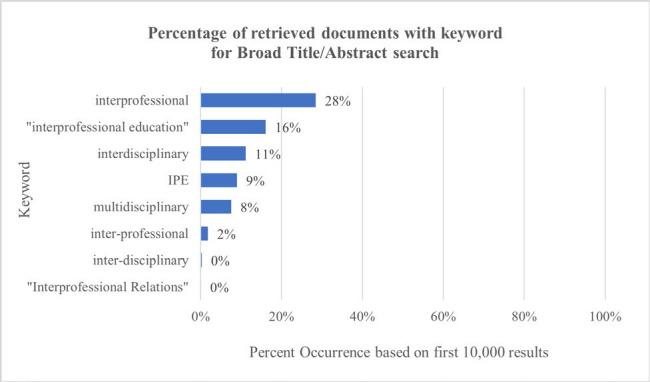
Frequency of individual terms

**Figure 4:**
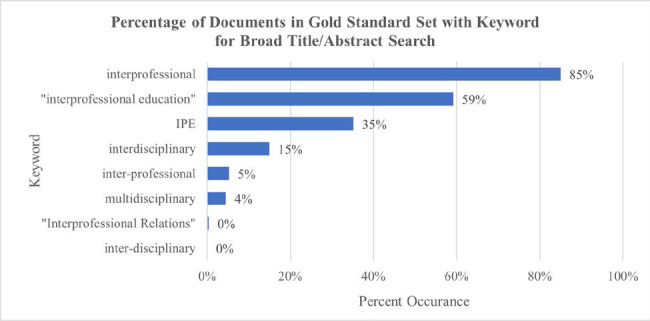
Recall of individual terms

There were three best performing hedges: a broad search strategy recommended for reviews and two narrow searches for efficient article discovery. The two narrower searches have 71.5% and 82.7% recall and 5,200 and 6,555 results in PubMed, respectively. The broader search has 95.1% recall while retrieving 55,791 results. Since the number of results returned by narrower searches was lower while still capturing most of the gold standard articles, these searches are more specific and focused than the other, broader keyword search. The broader keyword search achieved the best recall, however the number of results increased significantly, so it is at risk of also including more irrelevant articles. Therefore, the two narrower searches are recommended for quick retrieval of relevant papers while the broader keyword search is recommended for comprehensive literature reviews. These strategies, especially the broader keyword search, can be used in combination with additional search terms (e.g., terms for specific educational interventions) or other search filters to make the strategy more specific depending on research topics and literature searching needs.

While none of the search hedges reached 100% recall, the team determined that it was not possible to capture the missing studies even with the most sensitive version of the search, which captured 254 of 267 papers. Of the remaining papers, some lacked abstracts and so did not contain enough text to be captured through the keyword-based search approach and others did not contain any potential IPE terms in the title or abstract. While these papers were included in systematic reviews as relevant to IPE topics, this project did not follow the full-text screening process of a systematic review and so could not capture all papers that may include IPE terms in the full manuscripts. High quality systematic reviews, such as those used to source the gold standard set, employ other methods of searching (e.g., citation chaining, hand searching, grey literature searching, etc.), which cannot be replicated in a search hedge validation study limited to the PubMed search interface. While achieving 100% recall was the goal, other studies have also run into this issue [23] and the 95% recall achieved here is higher than in some other published validation papers.

A frequency analysis of the individual terms for IPE in published scholarship, showed that while Interprofessional Education is the established, modern term, there is variation on the terms used by authors in current scholarship and papers are still using outdated terms such as interdisciplinary or multidisciplinary to refer to interprofessional education. For example, in the first 10,000 search results from using the broad search hedge, interdisciplinary appears in 11.15% of retrieved documents, and multidisciplinary appears in 7.55%. The chart of search terms here can be chosen from in these use cases to find IPE papers.

## DISCUSSION

As there is exponential growth in the publication of IPE research, it has become more difficult to efficiently find relevant literature and stay abreast of all the latest research. This is an especially crucial issue for systematic reviews, which attempt to synthesize all of the available evidence with the purpose of informing clinical practice and future research [24,34]. To address this ongoing growth in research across disciplines, journals, and search databases, the results of this formal search hedge validation study provide recommended search terms for IPE studies, to improve future access to and synthesis of IPE research.

A complication in the search for IPE literature is how IPE search terms have changed over time. As societies change, so too, do terms and definitions [35], particularly in changing healthcare environments and contexts. Different terms such as interprofessional, interdisciplinary, multidisciplinary, and transdisciplinary may vary by professional type and field (e.g. social sciences versus medicine or nursing) [35]. These nuances are reflected in the literature. Even though the preferred term for IPE, interprofessional education, shows up in much of the literature, the authors' search-term level analysis of term frequency found that this phrase does not appear in all IPE scholarship even now. Furthermore, other, and older terms still need to be used to see all the scholarship and get to the level of recall needed for systematic reviews. Also, many articles do not use any recognizable phrase for IPE in the title, abstract, or author supplied keywords, meaning that researchers must rely on database indexing, context clues (e.g., the mention of more than one discipline in the abstract), or other searching methods (e.g., forward and backward citation searching) in order to find these papers.

Database indexing is an issue for IPE specifically, due to a lack of a specific, focused Medical Subject Heading (MeSH) term in PubMed until very recently. MeSH terms are used to index articles which refer to the same concepts but use slightly different terminology [15]. The phrase “Interprofessional Education” was not added as a subject heading in the MeSH database until 2021 [36]. Prior to this addition, relevant literature might have been categorized under the subject headings of “Education, Professional,” “Interprofessional Relations,” or “Interdisciplinary Communication,” none of which adequately and specifically describe IPE. These terms are all broad and conflate IPE with interprofessional collaboration or communication. While literature that is published from 2021 forward will have the IPE-specific MeSH term applied to their index terms, older literature is not retroactively re-indexed using the appropriate term. Therefore, it is important to combine keyword terms with MeSH to capture literature which uses a variety of terminology.

By contrast, researchers who do not need to run a comprehensive search for all IPE literature related to a population or intervention of interest, can use this identification of the frequency of terms for IPE in the literature to choose the best term or terms for their focused search. Researchers can select terms that are used most frequently by other scholars to find IPE papers and omit terms that are used less often, streamlining their search process. Overall, these results allow for recommendations to be made for an entire search hedge and for individual search terms for scholars who do not need an entire search hedge on IPE or who need a search with higher precision to find relevant papers.

Formally validating a search hedge, whether through relative recall or other methodologies, gives the research community an assessment of the performance of a search, so they can make an informed decision about if or how to use it to find relevant studies [12]. Using a formally validated search hedge allows researchers to save time in creating and testing their own search hedges. A past study on the time spent on systematic review tasks found that it took expert searchers an average of 8.4 hours to create and test a comprehensive literature search [37]. It can be assumed that it would take inexperienced searchers even longer to complete the process. Research on the quality of systematic reviews and meta-analyses has consistently found that many published reviews are lacking a rigorous search strategy, whether due to the volume of existing scholarship, a lack of expertise with the literature search process, a reluctance to take the time required for a comprehensive literature search, or all of the above [26]. Having a reliable, comprehensive search hedge already created and ready to use could save research teams a significant amount of time while helping ensure they do not miss important papers.

As Reeves and colleagues [38] note in their 2010 assessment of the evidence of IPE outcomes, “the evidence for the effects of IPE continues to rest on a variety of different IPE programs (e.g., in terms of learning activities, duration, and professional mix) and evaluation/research methods (experimental studies, mixed methods, qualitative studies) of variable quality.” Continued interest and investment in IPE has increased dramatically, and synthesis of this literature continues to be updated and expanded by international and national scholars [39]. One recommendation to lower the risk of overlooking relevant studies in reviewing IPE literature is increased awareness and use of reporting guidelines and exhaustive literature searches [39], such as the search hedge validation conducted within this study.

One limitation of this analysis is that it was completed prior to the National Library of Medicine's update to PubMed at the end of November 2022 which introduced proximity operators as an advanced search feature in the database. This search option was not available when the study was designed and carried out, so the search strategies tested and recommended in this paper do not include the use of proximity operators. While the addition of proximity operators would not change the relative recall of the broader, keyword search hedge, they may change the precision of the search and so should be tested in the future. Additionally, while the searches use terminology current as of the time of search testing, as IPE scholarship continues to grow in future years, the terminology used by researchers may change and require an update of this gold standard set and these search hedges.

Another limitation to be considered is this study, as in all bibliometric research, includes publication bias, the publication of only positive or significant results. The authors could only include published papers in the gold standard set used for the relative recall calculations, so nontraditional scholarship may not be accounted for. Also, the results of the search hedge validation depend on the original reviews' search strategies, since studies not included in the reviews which provided the gold standard set could not be used to test the search hedges. While reviews with low-quality methods were excluded in the screening phase, this is a general weakness of the relative recall methodology. The authors attempted to compensate for this known issue by creating a larger-than-normal gold standard set; validating the search strategy against more articles decreases the importance of any single, hypothetical, missing study [14].

Additionally, another potential limitation of the gold standard set is the ability for better-resourced scholars (be it financial or with increased research institutional support) to contribute more to IPE literature and their terminology choices to overinform the results here. This is not a problem specific to IPE scholarship, but a reflection of existing biases in academic publishing that are carried from primary research into secondary research [40,41].

Finally, while the authors present one search hedge with 95% relative recall, no search hedge in testing was able to achieve 100% recall. This is similar in resulting recall to past search hedge validation studies and comparable to Prady and colleagues [16] (92% recall), Ayiku et al. [11] (96%), and Golder et al. [23] (96%). In the end, the 95% recall achieved by the broad search hedge is still a high threshold and makes this search hedge suitable for systematic reviews. None of the three search hedges are a perfect research tool, but they will still be valuable to researchers.

In summary, this is the first study of its kind for IPE. It provides researchers with data on IPE search terms and search strategies through a relative recall validation of search strategies. These validated sets of search terms will make it easier and more efficient for scholars to find relevant IPE research in PubMed in the future. Next, the authors plan to translate these search hedges to the syntax of other MEDLINE platforms (e.g. Embase via OVID and Elsevier) and test the validation there, as other relative recall validation studies have achieved higher recall in Embase than in PubMed [20]. To remain current, it will also be important to continue to evaluate these search hedges over time, as IPE terminology and database indexing continue to evolve. Also, additional work in this area should test the precision, or sensitivity, of the IPE search hedges as relative recall methodology does not provide this measurement. For the present, these search hedges provide researchers with a range of customizable options for locating IPE scholarship in PubMed.

## Data Availability

The complete citation information for the gold standard set of references used for relative recall calculations, and the list of systematic reviews from which the gold standard set was derived is available in the Carolina Institutional Repository at https://cdr.lib.unc.edu/concern/data_sets/0c483v131. The tables and figures presented in this paper, including a document with the complete search hedges, are also available in the Carolina Institutional Repository at https://cdr.lib.unc.edu/concern/multimeds/ht24ww38q.
